# Serum ZAG Levels Were Associated with eGFR Mild Decrease in T2DM Patients with Diabetic Nephropathy

**DOI:** 10.1155/2017/5372625

**Published:** 2017-03-02

**Authors:** Lingling Xu, Weihong Yu, Meng Niu, Caixia Zheng, Bin Qu, Yan Li, Jing Wang, Ping Huang, O. Wang, Fengying Gong

**Affiliations:** ^1^Key Laboratory of Endocrinology of National Health and Family Planning Commission, Department of Endocrinology, Peking Union Medical College Hospital, Chinese Academy of Medical Science and Peking Union Medical College, Beijing 100730, China; ^2^Department of Ophthalmology, Peking Union Medical College Hospital, Chinese Academy of Medical Science and Peking Union Medical College, Beijing 100730, China; ^3^Department of Endocrinology, Traditional Chinese Medicine Hospital of Muping District of Yantai City, Yantai, Shandong, China; ^4^Department of Ophthalmology, Traditional Chinese Medicine Hospital of Muping District of Yantai City, Yantai, Shandong, China

## Abstract

*Objective.* To investigate the changes of serum zinc-*α*2-glycoprotein (ZAG) in type 2 diabetes mellitus (T2DM) with eGFR mild decrease. *Subjects and Methods.* A total of 438 T2DM patients (61.3 ± 4.0 y) were recruited and the demographic, anthropometric, and biochemical parameters were all collected. Serum ZAG levels were determined by commercially available ELISA kits. *Results*. The proportion of T2DM patients with the high tertile ZAG levels was 11.9% higher in patients with mildly decreased estimated glomerular filtration rate (eGFR) (<90 mL/min/1.73 m^2^) than those with the low tertile ZAG levels (*P* = 0.038). The probability of the eGFR < 90 mL/min/1.73 m^2^ in patients with the high ZAG levels was 94% higher than those with the low serum ZAG levels after adjusting for age, gender, and education [OR = 1.94, 95% CI (1.17–3.23), *P* = 0.0094]. This phenomenon was more likely to be observed in the condition of uACR ≥ 2.7 mg/mmol, WC ≥ 90 cm for men, or WC ≥ 85 cm for women. *Conclusion*. Serum ZAG levels were firstly found to be related with eGFR in T2DM patients. The patients with the high tertile ZAG levels were more likely to have mildly eGFR decrease, especially for female patients with higher uACR and bigger WC.

## 1. Introduction

Diabetes is one of the major risk factors for kidney damage, and people with diabetes have significantly increased risk for chronic kidney disease (CKD). Adipocytokines, which are linked to insulin resistance, low grade inflammation, endothelial dysfunction, and vascular damage, have been proposed as additional molecules able to modulate kidney function [1].

Zinc-*α*2-glycoprotein (ZAG), a newly identified 43 kDa adipocytokine, has been reported to play an important role in modulating glucose and lipid metabolism in adipose tissue, and it is closely associated with obesity and related disorders, such as diabetes and hypertension. Recently, a growing body of evidence showed that circulating ZAG levels were significantly lower in the patients with impaired glucose tolerance (IGT) and patients with newly diagnosed type 2 diabetes mellitus (T2DM) than age and gender matched controls [2]. ZAG levels were inversely correlated with body mass index (BMI), waist-to-hip ratio (WHR), the percentage of body fat, triglyceride (TG), fasting plasma glucose (FPG), fasting insulin, and homeostasis model assessment of insulin resistance (HOMA-IR) [2]. The administration of ZAG in mice dramatically countered some of the metabolic disorders of *ob/ob* and high-fat-diet- (HFD-) induced obese mice, including a reduction of body weight, fat mass, serum glucose, and insulin levels, and an improved response in the glucose tolerance test [3–5]. However, silencing ZAG in primary human adipocytes resulted in the reduction of the expression of insulin receptor substrate 1 (IRS1), glucose transporter type 4 (GLUT4), and peroxisome proliferator-activated receptor-γ coactivator-1 (PGC1*α*) [6].

Furthermore, recent several studies demonstrated that ZAG may be a novel urinary biomarker for normo-albuminuric diabetic nephropathy [7]. A study performed by Rao et al. indicated that ZAG was the second most abundant urinary protein in T2DM with diabetic nephropathy, and it was progressively elevated from normoalbuminuria to macroalbuminuria [8]. Subsequently, accumulated evidence showed that ZAG levels were closely associated with renal function [9, 10]. Serum ZAG levels were almost 2-fold higher in chronic kidney disease (CKD) 5, chronic hemodialysis (CH), and peritoneal dialysis patients as compared with controls [11]. Plasma from uremic patients could directly stimulate the production of ZAG in 3T3-L1 adipocytes in comparison with that from normal subjects [12]. However, there is no report about the changes of serum ZAG in diabetic nephropathy till now.

Therefore, in our current study, a total of 438 T2DM patients were recruited from north China, and serum ZAG levels were measured by ELISA methods. The results showed that serum ZAG levels were significantly associated with estimated glomerular filtration rate (eGFR) mild decrease in T2DM patients. The patients with high tertile ZAG levels were more likely to have mildly eGFR decrease, especially for female patients with higher urinary albumin-to-creatinine ratio (uACR) and bigger waist circumference (WC).

## 2. Subjects and Methods

### 2.1. Subjects

This cross-sectional study included patients with T2DM who were followed in the outpatient clinic at the Traditional Chinese Medicine Hospital of Yantai City's Muping District in Shandong, China, from October 2012 to May 2013. Patients were diagnosed with T2DM if they (1) were diagnosed with diabetes over 30 years of age, (2) had no history of ketoacidosis, (3) did not require insulin during the first three years following their diagnosis of diabetes, and (4) had eGFR ≥ 60 mL/min/1.73 m^2^.

The exclusion criteria included (1) impaired renal function (eGFR < 60 mL/min/1.73 m^2^); (2) history of hepatic dysfunction, acute kidney deterioration, clinical edema, skeletal muscle atrophy, pleural effusion or ascites, malnutrition, amputation, heart failure, malignant tumor, fracture within the past year, osteoporosis, thyroid disease, parathyroid disease, or another endocrine disease; (3) taking medications including androgens, progestins, and glucocorticoids, which were reported to regulate ZAG gene expression, in the past three months prior to enrollment [13–15]. The study was conducted in accordance with the Declaration of Helsinki. Approval for the study was obtained from the local ethics committee of the Department of Scientific Research at the Traditional Chinese Medicine Hospital of Yantai City's Muping District in Shandong. Informed consents were obtained from all patients prior to participation.

### 2.2. Clinical and Biochemical Assessments

Each patient participated in an interview in person to collect basic demographic data, history of T2DM and its complications, lifestyle, and past medical history. Interview data collected included age, gender, and time since diabetes diagnosis (TSDD), and current medications, including oral hypoglycemic drugs, insulin, antihypertensive drugs, and statins, were also collected.

Anthropometric data was collected through physical examination. Resting blood pressure (BP), height (Ht), weight, and waist circumference (WC) were all collected. Each measurement was made in duplicate and averaged. Body mass index (BMI) was calculated as the weight in kilograms divided by the square of height in meters.

Patients met a diagnosis of hypertension if they had at least one of the following criteria: (1) systolic blood pressure ≥ 140 mmHg, (2) diastolic blood pressure ≥ 90 mmHg, or (3) self-reported use of an antihypertensive medication.

Basal blood and urine samples were taken after 8–12 hours overnight fast as previously described [16]. Fasting plasma glucose (FPG), total cholesterol (TC), triglyceride (TG), hemoglobin A1c (HbA1C), and urinary albumin-to-creatinine ratio (uACR) were measured by routine-automated laboratory methods [16]. Albuminuria was defined as uACR ≥ 2.7 mg/mmol.

Serum levels of 25-hydroxy vitamin D [25(OH)D] were measured using an automated Roche electrochemiluminescence system (E170; Roche Diagnostics, Basel, Switzerland). Coefficients of variation (CV) for intra- and interassay were 1.7–7.8% and 2.2–10.7%, respectively. Serum ZAG levels were determined by commercially available human zinc-alpha2-glycoprotein ELISA kits according to the manufacturer's instruction (Cloud-Clone Corp, Houston, USA). The intra- and interassay CV for ZAG were 12.2 and 17.5%, respectively. eGFR was calculated using the Cockcroft-Gault equation: [(140 − age (years)) × body weight (kg)/72 × serum creatinine (mg/dl)] × 0.85 (if female). In this study, the patients were classified according to their eGFR values (in ml/min/1.73 m^2^) into the first two CKD stages as per the National Kidney Foundation Kidney Disease Outcomes Quality Initiative guidelines: normal or CKD stage 1: eGFR ≥ 90 mL/min/1.73 m^2^; CKD stage 2: eGFR 60–89 mL/min/1.73 m^2^ [17].

### 2.3. Statistical Analysis

All T2DM patients were classified into the high, median, and low groups according to their serum ZAG levels (about 33.3% for each tertile). Categorical variables are presented as absolute and relative values (%). Categorical variables were compared using chi-squared tests. Multivariate odds ratios (OR) and 95% confidence interval (CI) were derived from unconditional logistic regression models after adjusting for age (<61 years versus ≥61 years), gender, education (≤6 years versus >6 years), BMI (<24 kg/m^2^ versus ≥24 kg/m^2^), WC (men < 90 cm versus ≥90 cm, women < 85 cm versus ≥85 cm), hypertension (none versus yes), TC (<5.2 mmol/L versus ≥5.2 mmol/L), TG (<1.7 mmol/L versus ≥1.7 mmol/L), HbA1C (<7.0% versus ≥7.0%), FPG (<7.0 mmol/L versus ≥7.0 mmol/L), uACR (<2.7 mg/mmol versus ≥2.7 mg/mmol), vitamin D (≤50 nmol/L versus >50 nmol/L) [18], and eGFR (≥90 mL/min/1.73 m^2^ versus <90 mL/min/1.73 m^2^). Data was analyzed using SAS version 9.3 (SAS Institute Inc., Cary, NC), and the significance tests were two-tailed with *α* = 0.05.

## 3. Results

A total of 438 T2DM patients (mean age: 61.3 ± 4.0 y, male/female: 151/287) were recruited in this study. The demographic, clinical, and biochemical characteristics of all patients were detailed in Table 1. On the average, mean BMI was 27.3 ± 4.4 kg/m^2^, while mean WC was 98.5 ± 8.8 cm. As shown in Table 1, serum creatinine levels among these patients were all in normal range (73.3 ± 13.7 *μ*mol/L), while the median of eGFR was 89.7 mL/min/1.73 m^2^ (range from 74.7 to 97.2 mL/min/1.73 m^2^). Serum ZAG levels ranged from 1.13 to 1.60 *μ*g/ml.

Next, ZAG levels were categorized into tertiles (low, 0.57~1.19 *μ*g/ml; median, 1.19~1.52 *μ*g/ml; and high, 1.52~3.26 *μ*g/ml) as presented in Table 2. The proportion of patients with the high tertile ZAG levels was 9.1% and 11.9%, respectively, higher in patients with high TC levels (≥5.2 mmol/L, *P* = 0.045) and mildly decreased eGFR (<90 mL/min/1.73 m^2^, *P* = 0.038) than those with the low tertile ZAG levels. Nevertheless, the proportion of patients with the high serum ZAG levels was 17.0% lower in patients with low 25OHD levels (≤50 nmol/L, *P* = 0.0001) in comparison with those with the low serum ZAG levels. The similar phenomenon of the high ZAG levels decreased proportion was also observed in women, 12.9% lower than men with the low ZAG levels. However, there were no significant differences found between the proportions of patients with the high and low serum ZAG levels in referring to BMI, WC, and TG parameters although a different trend was demonstrated in Table 2 (*P* = 0.066,  0.097,  and 0.080, resp.).

In order to further investigate the relationship between eGFR and serum ZAG levels, unconditional logistic regression analysis was used, and eGFR was defined ≥90 mL/min/1.73 m^2^ as 0 and <90 mL/min/1.73 m^2^ as 1. As shown in Table 3, the probability of the eGFR < 90 mL/min/1.73 m^2^ in patients with the high ZAG levels was 94% higher than those with the low serum ZAG levels [OR = 1.94, 95% CI (1.17–3.23), *P* = 0.0094] after adjusting for age, gender, and education (Model 1). This increased probability of the eGFR < 90 mL/min/1.73 m^2^ still remained after further adjusting BMI, WC, hypertension, TC, TG, HbA1_C_, and FPG based on Model 1 [Model 2, OR = 1.87, 95% CI (1.09–3.22), *P* = 0.0228] and uACR and 25OHD based on Model 2 [Model 3, OR = 1.90, 95% CI (1.09–3.32), *P* = 0.0233].

**Figure 1:**
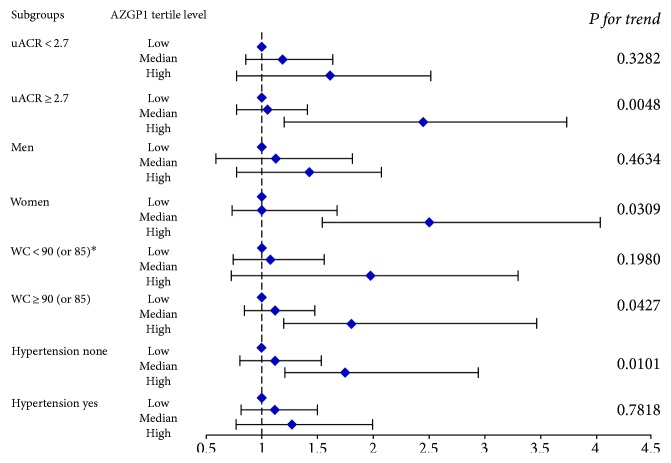
Further logistic regression analysis of eGFR in T2DM patients with the lowest, middle, and highest serum ZAG levels in subgroups analyses. Multivariate odds ratios (OR) and 95% confident interval (CI) from unconditional logistic regression models were used in the analysis, adjusted for age group (<61 y, ≥61 y), gender (men, women), BMI (<24, ≥24), ^∗^WC (<90, ≥90) in men and (<85, ≥85) in women, hypertension (none, yes), TC (<5.2, ≥5.2), TG (<1.7, ≥1.7), HbA1C (≤7.0, >7.0), FBG (<7.0, ≥7.0), uACR (<2.7, ≥2.7), and 25OHD VitD (>50 or ≤50). Stratified variables were also adjusted for in the subgroup analysis when possible.

Finally, the detailed logistic regression subgroup analysis of the association between the tertile ZAG levels and eGFR was further conducted after adjusting for age, gender, BMI, WC, TC, TG, HbA1_C_, FBG, uACR, and 25OHD. As demonstrated in Figure 1, T2DM patients with the high ZAG levels in our present study were more likely to have eGFR < 90 mL/min/1.73 m^2^ than those with the low ZAG levels in the condition of uACR ≥ 2.7 mg/mmol, WC ≥ 90 cm for men or WC ≥ 85 cm for women, especially for women without hypertension.

## 4. Discussion

Diabetic nephropathy is a microvascular complication associated with diabetes. It is an obvious risk factor for diabetes to result in slow deterioration of kidneys and, finally, lead to end-stage renal disease (ESRD). Because the course of diabetic nephropathy is chronic and irreversible, it is very essential to prevent the progression to ESRD by an early detection. ZAG, a novel adipocytokine mainly derived from the adipose tissue, has been reported earlier to be associated with glomerular disease [19]. Recent studies performed by Rao et al. demonstrated that urine ZAG levels were progressively increased across three categories of diabetic patients with normo-, micro-, and macroalbuminuria by a robust 2-D DIGE approach coupled with LCMS/MS in Indian T2DM patients, indicating that it is positively related with diabetes nephropathy progression [8]. Moreover, studies performed by Jain et al. also indicated the appearance of ZAG in the albumin-negative urine samples subsequently preceded the appearance of albumin in T2DM patients of South Asian Indians, suggesting that ZAG may be an earlier novel urinary biomarker useful for the screening of nonalbuminuric diabetic nephropathy [20]. Interestingly, a similar study performed on Singapore Chinese T2DM patients using the urinary proteomics approaches found that urinary ZAG levels were significantly higher in T2DM patients with persistently normal renal function (eGFR > 60 ml/min 1.73 m^2^ and urinary albumin-creatinine ratio < 30 mg/g) than those with mildly renal dysfunction (consistently, eGFR ≤ 60 mls/min 1.73 m^2^ and urinary albumin-creatinine ratio < 30 mg/g) (arbitrary unit ± standard error, 12.7 ± 1.5 versus 6.6 ± 1.3, *P* = 0.009), implying that urinary ZAG level is also increased in diabetes patients with normoalbuminuria when they only have eGFR less than 60 ml/min 1.73 m^2^ [7]. All of these studies suggest that urine ZAG levels not only would be an earlier biomarker for diabetic nephropathy but also are associated with the renal dysfunction. In line with these results, we firstly found in our present study that the proportion of patients with the high serum ZAG levels was higher in patients with mildly decreased eGFR (<90 ml/min) than those with the low serum ZAG levels, and the probability of the eGFR < 90 ml/min in patients with the high ZAG levels was 94% higher than those with the low serum ZAG levels after adjusting for age, gender, and education. This phenomenon was more likely to happen in female patients with higher uACR (≥2.7 mg/mmol) and bigger WC (≥90 cm for men or ≥85 cm for women). This result suggests that serum ZAG levels (in addition to urine ZAG levels) are more likely to be increased in female T2DM patients with diabetic nephropathy even if they only have mildly decreased eGFR (<90 ml/min), an earlier predictor than what is reported in previous report (eGFR less than 60 ml/min 1.73 m^2^) [7].

Recently, a few studies showed that serum ZAG levels were evidently elevated in chronic hemodialysis (CH) patients, suggesting a decrease of its renal clearance [9, 10, 21]. One study performed in the adult patients with chronic kidney disease (CKD) also demonstrated that ZAG concentration sharply increased in CKD 5, CH, and peritoneal dialysis patients, implying that the kidney could play an important role in the maintenance of serum ZAG levels [11]. Normally, a fragment of serum ZAG passes through the glomerular membrane after which it is completely reabsorbed at the tubular level due to its rather small molecular weight (43 kDa) and size (Stokes radius 3.24 nm). Therefore, the renal function impairment with decreased GFR results in a rise in ZAG/creatinine clearance ratio [22], as what we observed in our present investigation in T2DM with mildly eGFR decrease. Another explanation for the increased serum ZAG concentration in the study may come from the increased secretion and production of ZAG from epithelia cells from liver, kidney, breast, sweat glands, and gastrointestinal tract, as well as white adipose tissue (WAT). Pelletier et al. reported that the protein content of ZAG in subcutaneous WAT from patients with CKD was notably increased (5.7-fold) compared to age-matched controls, and the nephrectomized rodents exhibited greatly upregulated ZAG levels in WAT associated with significant decrease in WAT deposition [12]. Gohda et al. reported that the ZAG mRNA levels in the liver and kidney were significantly increased in KK/Ta mouse, a spontaneous animal model of type 2 diabetes [23]. Selva et al. demonstrated that there was a significant positive correlation between ZAG serum levels and mRNA levels of ZAG in subcutaneous adipose tissue (SAT), visceral adipose tissue (VAT), and liver, implying that both adipose tissue and liver seem to be the important contributors to ZAG systemic levels [24]. Taking all of these studies into consideration, serum ZAG concentration seems to be influenced by both the renal elimination and the secretion from organism tissues. Furthermore, markers of renal function such as eGFR should be considered in studies investigating the physiology and regulation of ZAG.

The physiologic significance of increased ZAG serum concentrations in T2DM with mildly eGFR decrease remains to be elucidated. It has been reported that ZAG is closely linked with obesity and obesity-related disorders such as diabetes, hypertension, and dyslipidemia [3, 25, 26]. Yeung et al. demonstrated that serum ZAG levels were markedly elevated in overweight/obesity, hypertension, hypertriglyceridemia, or T2DM in south Chinese subjects, and serum ZAG also significantly associated the obesity-related parameters (WC and BMI), insulin resistance (increased fasting insulin, increased HOMA-IR), and increased TG levels [27]. Studies performed by Olofsson et al. showed that serum levels of ZAG were significantly correlated with serum TC and TG levels, and a polymorphism in the ZAG gene was also associated with circulating TC levels in healthy and obese Swedish population, suggesting that ZAG is involved in lipid metabolism [28]. The similar results were observed in HIV-1-infected patients which showed that serum ZAG levels were in close relationship with TC and high-density lipoprotein cholesterol (HDLc) [29]. In good agreement, we observed in the present study that the proportion of T2DM patients with high TC (≥5.2 mmol/L) was higher in the high ZAG level group than in the low ZAG levels group, suggesting T2DM patients with high TC had higher ZAG levels. The similar association trend was also observed between ZAG and TG, BMI, and WC levels although *P* value was higher than 0.05. These lines of clinical evidence imply that the elevation of serum ZAG in T2DM patients with mildly eGFR decrease might be a compensatory upregulation of the human body to counteract the metabolic disorders imposed by T2DM.

It is also of interest in our current study to note that the proportion of T2DM patients with the highest serum ZAG levels in men was 12.9% higher than women with the lowest ZAG levels, suggesting that male T2DM patients were more likely to have higher serum ZAG levels than females. The same phenomenon was observed in a Hong Kong Chinese population study performed by Yeung et al. which showed that serum ZAG levels in men were 72.5% higher than in women [27]. Our recent unpublished data also demonstrated that men had higher serum ZAG levels than women both in normal weight controls and in overweight/obese patients. A possible explanation for this gender-based discrepancy is that ZAG may have sex-specific regulation or different body fat percentage and distribution in women and men.

To the best of our knowledge, we firstly reported that the proportion of T2DM patients with the high serum ZAG levels was lower in patients with low 25OHD levels than those with the low serum ZAG levels, suggesting that T2DM patients with the high serum ZAG levels were more likely to have lower 25OHD levels. Up to now, only two literatures reported the similar changes of ZAG and vitamin D-binding protein in serum of newly diagnosed multiple myeloma patients [30] and saliva obtained from generalized aggressive periodontitis [31]. Further studies are needed to be done to address the relationship between ZAG and vitamin D.

In conclusion, in our present study, serum ZAG levels were firstly found to be correlated with eGFR in T2DM patients. The patients with higher ZAG levels were more likely to have mildly eGFR decrease, especially for female patients with higher uACR and bigger WC. The main limitation of our study is that it is a cross-sectional observational study and therefore that it is difficult to make causal inference. The participants of this study came from a single hospital, and our findings may not be generalizable to other populations. Further prospective studies are needed to better elucidate the potential role of ZAG in T2DM.

## Figures and Tables

**Table 1:** Clinical and biochemical characteristics of the study participants (*N* = 438) [mean ± SD or median (interquartile range)].

Items	Values	Items	Values
Age (y)	61.3 ± 4.0	Creatinine (*μ*mol/L)	73.3 ± 13.7
Gender (M : F)	151 : 287	uACR (mg/mmol)	33.5 (0–604)
Education (y)	6 (0–18)	eGFR (mL/min/1.73 m^2^)	89.7 (74.7–97.2)
BMI (kg/m^2^)	27.3 ± 4.4	FPG (mmol/L)	9.4 ± 3.1
WC (cm)	98.5 ± 8.8	PPG (mmol/L)	12.8 ± 3.9
SBP (mmHg)	137 ± 15	HbA1C (%)	8.0 ± 1.7
DBP (mmHg)	83.7 ± 8.4	TG (mmol/L)	1.47 (0.37 to 46.0)
25OHD (nmol/L)	45.0 ± 17.8	TC (mmol/L)	5.68 ± 1.32
ZAG (*μ*g/ml)	1.46 (1.13 to 1.60)		

BMI: body mass index; WC: waist circumference; SBP: systolic blood pressure; DBP: diastolic blood pressure; uACR: urinary albumin-to-creatinine ratio; eGFR: estimated glomerular filtration rate; FPG: fasting plasma glucose; PPG: postprandial plasma glucose; TG: triglyceride; TC: total cholesterol.

**Table 2:** Characteristics of T2DM patients with the low, middle, and high serum ZAG levels.

	ZAG (*μ*g/ml)				
	Total	Low (0.57~1.19)	Median (1.19~1.52)	High (1.52~3.26)	*P*
Age (y)					
<61	198 (45.2)	66 (45.5)	66 (44.9)	66 (45.2)	
≥61	240 (54.8)	79 (54.5)	81 (55.1)	80 (54.8)	0.95
Gender					
Men	151 (34.5)	36 (24.8)	60 (40.8)	55 (37.7)	
Women	287 (65.5)	109 (75.2)	87 (59.2)	91 (62.3)	**0.022**
Education (y)					
≤6	205 (46.8)	71 (49.0)	74 (50.3)	60 (41.1)	
>6	233 (53.2)	74 (51.0)	73 (49.7)	86 (58.9)	0.18
BMI (kg/m^2^)					
<24	61 (13.9)	16 (11.0)	18 (12.2)	27 (18.5)	
≥24	377 (86.1)	129 (89.0)	129 (87.8)	119 (81.5)	0.066
WC (cm)					
<90 (or 85)^1^	28 (6.4)	7 (4.8)	7 (4.8)	14 (9.6)	
≥90 (or 85)	410 (93.6)	138 (95.2)	140 (95.2)	132 (90.4)	0.097
Hypertension^2^					
None	302 (69.0)	92 (63.5)	105 (71.4)	105 (91.9)	
Yes	136 (31.0)	53 (36.5)	42 (28.6)	41 (28.1)	0.12
TC (mmol/L)					
<5.2	79 (18.0)	33 (22.8)	26 (17.7)	20 (13.7)	
≥5.2	359 (82.0)	112 (77.2)	121 (82.3)	126 (86.3)	**0.045**
TG (mmol/L)					
<1.7	307 (70.1)	109 (75.2)	102 (69.4)	96 (65.8)	
≥1.7	131 (29.9)	36 (24.8)	45 (30.6)	50 (34.2)	0.080
HbA1C (%)					
≤7.0	192 (43.8)	71 (49.0)	56 (38.1)	65 (44.5)	
>7.0	246 (56.2)	74 (51.0)	91 (61.9)	81 (55.5)	0.45
FPG (mmol/L)					
≤7.0	139 (31.7)	44 (30.3)	40 (27.2)	55 (37.7)	
>7.0	299 (68.3)	101 (69.7)	107 (72.8)	91 (62.3)	0.18
uACR (mg/mmol)					
<2.7	261 (59.6)	88 (60.7)	85 (57.8)	88 (60.3)	
≥2.7	177 (40.4)	57 (39.3)	62 (42.2)	58 (39.7)	0.94
25OHD (nmol/L)					
>50	135 (30.8)	25 (17.2)	60 (40.8)	50 (34.2)	
≤50	303 (69.2)	120 (82.8)	87 (59.2)	96 (65.8)	**0.0017**
eGFR (ml/min)					
≥90	176 (40.2)	64 (44.1)	65 (44.2)	47 (32.2)	
<90	262 (59.8)	81 (55.9)	82 (55.8)	99 (67.8)	**0.038**

^1^WC defined as (<90, ≥90) in men and (<85, ≥85) in women, all units in cm.

^2^Hypertension defined as SBP ≥ 140 mmHg and/or DBP ≥ 90 mmHg, or self-reported use of an antihypertensive medication.

**Table 3:** Unconditional logistic regression analysis of eGFR in T2DM patients with the lowest, middle, and highest serum ZAG levels.

	ZAG_3 level OR (95% CI)			
	Low	Median	High	*P for trend*
Number of subjects	145	147	146	
Model 1	1.00	1.10 (0.67, 1.80)	1.94 (1.17, 3.23)	**0.0094**
Model 2	1.00	1.18 (0.70, 1.99)	1.87 (1.09, 3.22)	**0.0228**
Model 3	1.00	1.22 (0.71, 2.10)	1.90 (1.09, 3.32)	**0.0233**

Multivariate odds ratios (OR) and 95% confident intervals (CI) from unconditional logistic regression models were used in the analysis.

Model 1: basic model, adjusted for age group, gender, and education.

Model 2: further adjusted for BMI (<24 kg/m^2^, ≥24 kg/m^2^), WC (<90 cm, ≥90 cm) in men and (<85 cm, ≥85 cm) in women, hypertension (none, yes), TC (<5.2 mmol/L, ≥5.2 nnol/L), TG (<1.7 mmol/L, ≥1.7 mmol/L), HbA1_C_ (≤7.0%, >7.0%), FPG (≤7.0 mmol/L, >7.0 mmol/L), based on Model 1.

Model 3: full model, further adjusted for uACR (<2.7 mg/mmol, ≥2.7 mg/mmol) and 25OHD (>50 nmol/L, ≤50 nmol/L) based on Model 2.
